# Methyl Gallate and Amoxicillin-Loaded Electrospun Poly(vinyl alcohol)/Chitosan Mats: Impact of Acetic Acid on Their Anti-*Staphylococcus aureus* Activity

**DOI:** 10.3390/polym17010007

**Published:** 2024-12-24

**Authors:** Pimsumon Jiamboonsri, Weradesh Sangkhun, Sompit Wanwong

**Affiliations:** 1 Faculty of Medicine, King Mongkut’s Institute of Technology Ladkrabang, 1 Chalongkrung Road, Ladkrabang, Bangkok 10520, Thailand; 2 Materials Technology Program, School of Energy, Environment and Materials, King Mongkut’s University of Technology Thonburi, 126 Pracha Uthit Road, Bang Mod, Bangkok 10140, Thailand; weradesh.s@gmail.com (W.S.); sompit.wan@kmutt.ac.th (S.W.)

**Keywords:** methyl gallate, amoxicillin, poly(vinyl alcohol)/chitosan, electrospun nanofiber, anti-*S. aureus* material

## Abstract

Methyl gallate (MG), a natural phenolic compound, exhibits in vitro synergistic activity with amoxicillin (Amox) against methicillin-resistant *Staphylococcus aureus* (MRSA), a global health concern. This study developed electrospun nanofibers incorporating MG and Amox into a poly(vinyl alcohol) (PVA)/chitosan (CS) blend to target both methicillin-susceptible *S. aureus* (MSSA) and MRSA. The formulation was optimized, and the impact of acetic acid on antibacterial activity was evaluated using agar disc diffusion. The final formulation was fabricated and characterized using SEM, FTIR, DSC, swelling, and release behavior analyses to understand its antibacterial efficacy. Results revealed that acetic acid eliminated antibacterial activity, but MG (64 mg/mL) and Amox (2.5 mg/mL) were successfully incorporated into a PVA/CS solution prepared with deionized water. The resulting nanofiber mats featured nanoscale fibers (126 ± 45 nm) with and micron-oval beads. Despite the in vitro synergism, the MG/Amox/PVA/CS mats showed no significant improvement over MG or Amox alone against MRSA, likely due to their physicochemical properties. FTIR and DSC results confirmed molecular interactions between the active compounds and the polymer matrix, which may cause a minimal swelling and low drug release at 24 h. This study offers insights into the potential of MG/Amox-loaded nanofibers for anti-MRSA material development.

## 1. Introduction

Amox is a semisynthetic β-lactam antibiotic belonging to the class of aminopenicillins, characterized by the addition of an amino group to the benzylpenicillin structure [1,2]. Aminopenicillins were the first β-lactam antibiotics to demonstrate broad-spectrum activity against both Gram-negative and Gram-positive bacteria, including *Haemophilus influenzae*, *Escherichia coli*, *Salmonella* spp., *Shigella* spp., *Streptococcus* spp., and *Staphylococcus* spp. [1]. However, bacterial resistance has limited the clinical use of Amox due to the production of β-lactamases, which hydrolyze the β-lactam ring by splitting the amide bond in the antibiotic’s structure [3]. This breakdown prevents Amox from binding to the penicillin-binding protein (PBP), which is an enzyme essential for cell wall synthesis. To overcome this limitation, a second agent, either with or without antibacterial activity, is often combined with Amox to inhibit β-lactamase enzymes irreversibly. For example, clavulanate, a β-lactamase inhibitor with no intrinsic antibacterial activity, is combined with Amox to restore its efficacy against β-lactamase-producing strains of *H. influenzae*, *Moraxella catarrhalis*, and *S. aureus* [4,5]. Despite its widespread use, the emergence of bacterial resistance through various mechanisms has reduced the effectiveness of Amox-clavulanate, even though it remains one of the most frequently prescribed antibiotics in emergency and primary care settings worldwide [6]. This has spurred research into novel combinations with Amox to combat resistant bacterial infections. Our recent report showed that MG has a synergistic effect with only β-lactamase-unstable antibiotics, such as penicillin, ampicillin, and Amox, against MRSA [7,8]. Specifically, the addition of MG reduced the minimum inhibitory concentration (MIC) of Amox by fourfold against MRSA, although it showed no significant effect against MSSA [8]. The anti-bacterial mechanism of MG is proposed to involve disruption of the bacterial cell membrane and cell wall without targeting PBPs [8], and as a result, the combination of MG and β-lactam antibiotics showed no antagonistic effects against MSSA. Furthermore, MG has been shown to enhance antibiotic efficacy and restore bacterial susceptibility by overcoming resistance mechanisms including the inhibition of efflux pumps and the downregulation of quorum sensing and virulence genes involved in biofilm formation [9,10]. Therefore, the MG–Amox combination could be particularly advantageous for treating resistant bacterial infections. However, MG’s low bioavailability and susceptibility to hepatic enzyme metabolism limit its use for oral administration [11,12]. As a result, MG may be better suited for external or topical applications, making it a promising candidate for localized treatment strategies.

Electrospinning is an efficient technique used in the biomedical field to produce functional nanofibers from synthetic and natural polymers by applying high voltage to a polymer solution [13,14]. The process begins when the electrostatic force overcomes the surface tension of the solution, resulting in the formation of a Taylor cone at the nozzle tip. A charged polymer jet is subsequently ejected, undergoing rapid stretching and thinning due to electrostatic repulsion and solvent evaporation, which ultimately leads to the formation of nanometer-scale fibers [14].

CS, a derivative of chitin, is obtained from the shells of shrimp, crabs, and other arthropods [15]. Its chemical structure is a linear polysaccharide composed of β-(1→4)-linked D-glucosamine (deacetylated units) and N-acetyl-D-glucosamine (acetylated units) [16]. While CS’s structure imparts high viscosity, this characteristic poses challenges for electrospinning as the electrostatic field struggles to overcome the surface tension of the solution. Despite its electrospinning challenges, CS offers notable physicochemical and biological properties, including biodegradability, nontoxicity, and wound-healing acceleration. To enhance CS’s electrospinnability, it must be dissolved in an acid solution to optimize its viscosity or blended with another polymer.

Petrova et al. demonstrated the use of dilute 30% acetic acid as an electrospinning solvent to fabricate nanofibers from 3% CS, 4% sodium alginate, and 7.5% chitin nanowhiskers, resulting in uniform fibers with diameters of 200–300 nm and no bead formation [17]. Similarly, at a higher concentration of 70% acetic acid, CS/PVA blends could be electrospun at varying volume ratios (30/70, 50/50, and 70/30) under constant process parameters, producing uniform, bead-free fibers [18]. PVA, a synthetic polymer, is often chosen for electrospinning due to its chemical stability, temperature resistance, high mechanical strength, and excellent fiber-forming properties [19]. PVA is also widely studied in biomedical applications because of its nontoxicity, biocompatibility, and biodegradability. However, its water solubility can limit its applications [19], leading to its frequent blending with other polymers to achieve the desired properties in electrospun fibers. Several studies have successfully fabricated functional PVA/CS nanofibers incorporating various active compounds, including antibiotics [20]. For examples, hybrid nanofibers of PVA/CS loaded with silver and copper nanoparticles have been developed for wound dressing applications, demonstrating effective inhibition zones against both Gram-negative and Gram-positive bacteria [21]. Abbaspour et al. incorporated 40 wt% mafenide acetate, a sulfonamide antibiotic, into CS/PVA (70/30) nanofibers, enhancing their antimicrobial activity against *Pseudomonas aeruginosa* and *S. aureus* [22]. Additionally, PVA/CS nanofibers cross-linked with an aqueous glutaraldehyde solution were successfully loaded with ampicillin, and the release of ampicillin sodium from the cross-linked composite nanofibers followed the Fickian diffusion mechanism [23]. However, the antibacterial properties of these fabricated materials were not fully explored.

In this study, we aimed to develop PVA/CS nanofibers loaded with MG and Amox for anti-*S. aureus* applications. The impact of acetic acid and concentrations of MG and Amox were optimized by fabricating PVA/CS nanofibers and evaluating their antibacterial activity. The optimized electrospun mats were characterized using various techniques, including scanning electron microscopy (SEM), Fourier transform infrared spectroscopy (FTIR), and differential scanning calorimetry (DSC). Additionally, swelling and drug release studies were conducted to better understand their antibacterial properties against both MSSA and MRSA strains.

## 2. Materials and Methods

### 2.1. Materials

MG (>98%), Amox trihydrate (>98%), PVA (Lot no. 2GQYA, a polymerization degree, *n* = approx. 1700), and CS (Lot no. ETJOI) were obtained from Tokyo Chemical Industry (Tokyo, Japan). CS (MW 20–100 kDa), extracted and purified from crab shells (Crustacea), exhibited a viscosity of 5–20 mPa-s in 0.5% acetic acid solution at 20 °C and a deacetylation degree of 84.3% [24]. Acetic acid (glacial) was sourced from Merck (Darmstadt, Germany). Other chemicals and solvents were of analytical grade and obtained from local distributors.

### 2.2. Methods

#### 2.2.1. Preparation of Electrospinning Solutions

A stock polymer solution was prepared by dissolving 10 g of PVA and 0.1 g of CS in 100 mL of deionized (DI) water, with or without the addition of 20% *v/v* acetic acid (pH ≈ 2). The mixture, consisting of 10% *wt*/*v* PVA and 0.1% *wt*/*v* CS, was stirred and heated at 90 °C for approximately 3 h. Polymer solutions were completely dissolved in the presence of acetic acid. However, in the absence of acetic acid, the polymer solutions underwent a 3 h heating process forming the dispersed solution.

To optimize the concentration of active compounds and evaluate the impact of acetic acid on the anti-*S. aureus* activity of the fabricated nanofiber mats, the amounts of MG and Amox were calculated based on previous studies [8,25]. The formulations were adjusted according to the parameters outlined in Table 1. For example, to prepare 64 mg/mL MG/PVA/CS, 640 mg of MG was added to 10 mL of PVA/CS solution. The polymer solution was then ultrasonicated for 20 min before fiber fabrication via electrospinning. Summary concentrations of each compound in the formulation are listed in Table 1.

#### 2.2.2. Fabrication of Electrospinning Nanofibers

**Figure 1:**
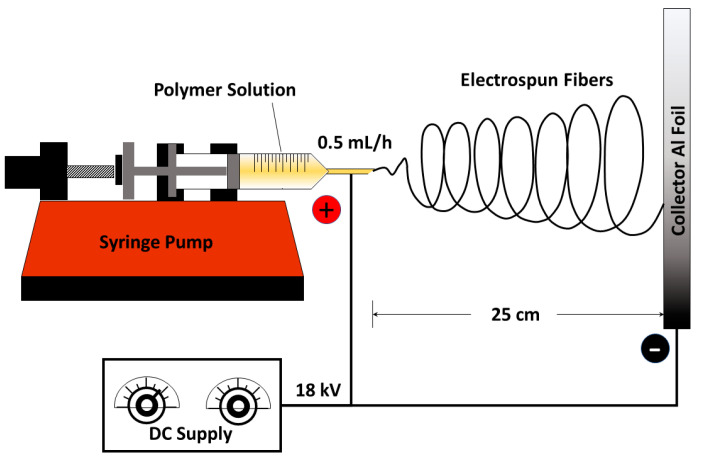
Schematic illustrations showing the electrospinning method to fabricate MG/Amox/PVA/CS nanofibers.

A total of 5 mL of each formulation was placed in a 10 mL plastic syringe fitted with an 18G blunt-tip needle. A positive electrical wire was connected to the syringe tip, and a negative wire was attached to the conductive collector. The electrospinning conditions were set as follows: 18G syringe tip, collector–tip distance of 25 cm, dispensing flow rate of 0.5 mL/h, and an applied DC voltage of 18 kV (Figure 1). Electrospinning was conducted at 80% relative humidity under atmospheric conditions, with the syringe positioned horizontally at a 0° angle.

#### 2.2.3. In Vitro Anti-*S. aureus* Activity

*S. aureus* ATCC 25923 (MSSA) and *S. aureus* ATCC 43300 (MRSA) were purchased from the American Type Culture Collection. The bacteria were stored at −80 °C in a mixture of tryptic soy broth (TSB; Becton Dickinson & Co., Sparks, MD, USA) and 20% *wt*/*v* glycerol until use. For experiments, both bacterial strains were grown separately in Mueller–Hinton broth (Becton Dickinson & Co., Sparks, MD, USA) at 37 °C for 24 h. Subsequently, the turbidity of the bacterial suspension was adjusted using a UV spectrophotometer (Thermo Scientific, GENESYS 10S, Waltham, MA, USA) at 600 nm to achieve an optical density (OD) of 0.1, corresponding to approximately 10⁷ colony-forming units (CFU/mL).

All test samples were cut into 1 × 1 cm^2^ pieces and pre-sterilized with ultraviolet light for 15 min before testing. The antibacterial activity of the nanofiber mats was evaluated using the disc diffusion method as described in our previous study [25]. Briefly, each prepared bacterial suspension was swabbed onto Mueller–Hinton agar (Becton Dickinson & Co., Sparks, MD, USA) and allowed to air dry at room temperature (25 °C). The pre-sterilized mats were then placed on the agar plates. After incubation at 37 °C for 24 h, the inhibition zone diameters (mm) were measured. Experiments were performed in triplicate. Neat PVA and PVA/CS mats were used as material controls. Additionally, Amox (10 μg/disc, 1 × 1 cm^2^) and a paper filter (1 × 1 cm^2^) were used as the positive and negative controls, respectively.

#### 2.2.4. Morphological, Chemical, and Thermal Characterizations of Nanofibers

The morphological characteristics of MG, Amox, and MG/Amox/PVA/CS nanofibers were observed using a field-emission SEM (FE-SEM; Versa 3D FEG). The fiber and bead diameters were measured using ImageJ software (version 1.53k). Chemical functionality of the nanofibers was analyzed using FTIR spectroscopy (Thermo Scientific, Nicolet 6700 spectrophotometer). FTIR spectra were recorded over a wavenumber range of 700–4000 cm^−1^. The thermal properties of the samples were evaluated using DSC (NETZSCH DSC 204 F1 Phoenix, NETZSCH-Gerätebau GmbH, Selb, Germany). The samples were heated from 50 °C to 250 °C at a rate of 5 °C/min.

#### 2.2.5. Swelling, Water Retention, and Weight Loss Test

The swelling behavior of the electrospun fibers was evaluated by immersing fiber samples (1.5 × 1.5 cm^2^) in DI water (pH 7, 30 ± 1 °C) for 24 h. Prior to the test, the original weight of each fiber sample was recorded. After immersion, excess surface water was gently removed using filter paper, and the weight of the swollen fibers was measured. The degree of swelling was determined using the Equation (1).

To evaluate water retention and weight loss, the swollen sample was placed in a fume hood at room temperature (open air, ~60% relative humidity) for 24 h, after which its weight was recorded. The sample was then dried in a vacuum oven at 60 °C for 24 h, and its weight was measured again. Water retention and weight loss were determined by using Equations (2) and (3) [26], respectively:

(1) $$Degree\;of\;Swelling\;(\%)=\frac{W_{s}-W_{0}}{W_{0}}\times 100$$

(2) $$Water\;Retention\;\left(\%\right)=\frac{W_{t}-W_{d}}{W_{s}-W_{d}}\times 100$$

(3) $$Weight\;loss\left(\%\right)=\frac{W_{0}-W_{d}}{W_{0}}\times 100$$

where W_s_, W_d_, W_t_, and W_0_ represent weights of the swollen sample, the weight of the dried sample, the weight of the swollen sample after 24 h of air exposure, and the original weight of the sample, respectively. Each formulation was tested in triplicate (*n* = 3).

#### 2.2.6. Release Test

Specimens (1.5 × 1.5 cm^2^, approximately 40 mg initial weight, *n* = 3) were immersed in 10 mL of DI water for 24 h. After the incubation period, the samples were collected, and the absorbed solvent within the fibers was gently squeezed out. The resulting solution was filtered using a 0.45 µm hydrophilic PTFE syringe filter. The released concentrations of Amox and MG were determined using a UV-visible spectrophotometer (Thermo Scientific, GENESYS 10S, Waltham, MA, USA) at wavelengths of 230 nm and 270 nm, respectively.

The linear regression equation for MG and Amox in the concentration range of 0.5–50 µM were Y = 10129X − 0.0024 for MG and Y = 10025X + 0.0136 for Amox with an r^2^ of 0.9999. The released concentration of each compound was converted to the released amount based on their respective molecular weights. The releasing percentage was reported using the Equation (4):

(4) $$Release(\%)=\frac{w_{Release}}{w_{int}}\times 100$$

where W_release_ and W_int_ are the releasing amount and initial amount for each compound, respectively.

#### 2.2.7. Statistical Analysis

Statistical analyses of the antibacterial test results were conducted using SPSS software (version 29.0, SPSS Inc., Chicago, IL, USA). Analysis of variance (ANOVA) was performed to evaluate the data, and significant differences between means were determined using either Tukey’s honestly significant difference (HSD) test or Dunnett’s T3 test at a significance level of *p* < 0.05.

## 3. Results and Discussion

### 3.1. Optimization Concentrations of Active Compounds: Impact of Acetic Acid on the Anti-S. aureus Activity of Electrospun Mats

**Figure 2:**
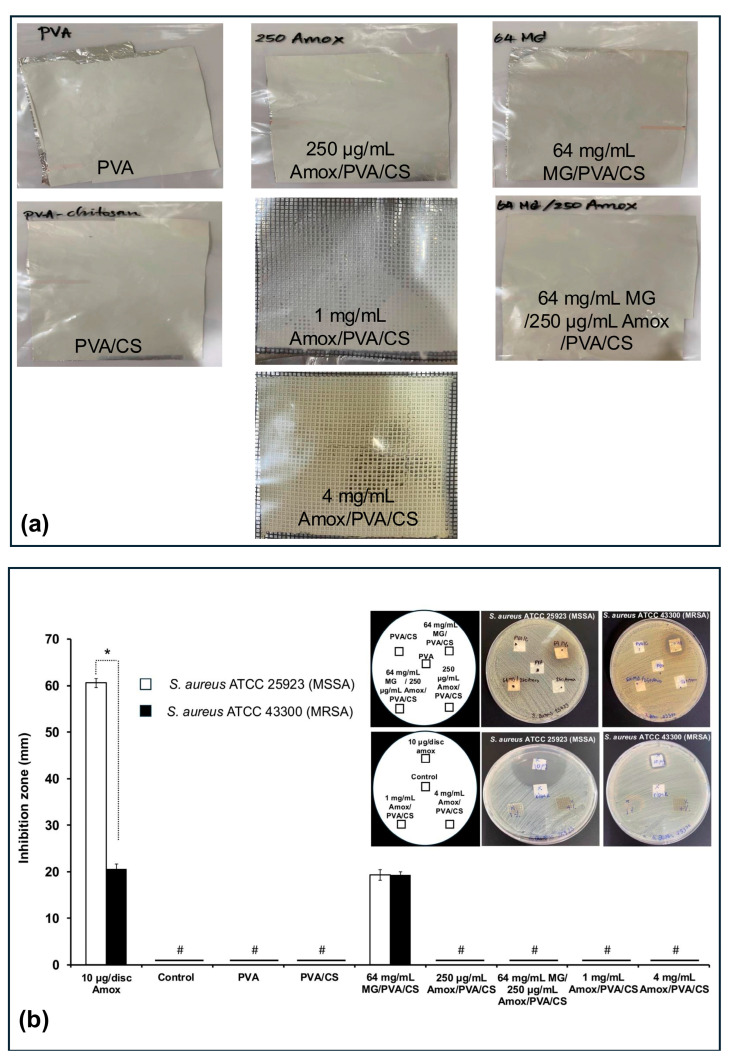
(**a**) Images of the electrospun nanofiber mat fabricated under optimization conditions with acetic acid conditions (formulation set A) and (**b**) their antibacterial activities compared with neat PVA and PVA/CS against *S. aureus* ATCC 25923 (MSSA) and 43300 (MRSA). Each symbol indicates the mean ± S.D. (*n* = 3). # The inhibition zone could not be determined. * Denotes a significant difference between the mean inhibition zones of the two bacterial strains (*p* < 0.05).

According to the literature, acetic acid is commonly used to solubilize CS and PVA for film production or nanofiber fabrication [27,28,29]. In this study, 10% *wt*/*v* PVA and 0.1% *wt*/*v* CS were dissolved in 20% *v/v* acetic acid before incorporating active compounds in formulation set A. The PVA/CS electrospun mats loaded with 64 mg/mL MG and/or 250 μg/mL Amox exhibited a white, opaque appearance similar to the neat mats (Figure 2a). The antibacterial activities of the mats are presented in Figure 2b. For the neat PVA or PVA/CS mats, no inhibition zones were observed against either MSSA or MRSA strains, indicating the absence of anti-*S. aureus* activity. This result aligns with previous reports, which found that pure 5 wt% PVA or 1 wt% CS films did not exhibit antibacterial properties [30,31]. However, blending higher concentrations of CS (4 wt%) with PVA (10 wt%) demonstrated a partial reduction in bacterial growth under the specimens on agar plates [30,32].

For the PVA/CS mats loaded with active compounds, only 64 mg/mL MG/PVA/CS exhibited antibacterial activity, forming inhibition zones of approximately 19 mm against both MSSA and MRSA. In contrast, 250 μg/mL Amox/PVA/CS exhibited no antibacterial activity against either strain. The results indicated that the loading concentration of Amox may be lower than the effective concentration. Furthermore, the incorporation of both MG and Amox at similar concentrations also showed no antibacterial activity. This result may indicate an interaction among active compounds and polymers that hinders MG diffusion onto the agar plate, thereby reducing its ability to inhibit bacterial growth despite potential synergistic effects [8].

To determine an effective concentration of Amox, its loading concentration was increased to 1 and 4 mg/mL in acidic polymer solution. A mesh substrate was used to enhance solvent evaporation and drug diffusion through its open structure. The fabricated mats with 1 mg/mL Amox appeared white and opaque, while 4 mg/mL mats displayed a pale-yellow color (Figure 2a). As shown in Figure 2b, despite the increased Amox concentration, neither formulation inhibited MSSA or MRSA growth. In contrast, 10 μg/disc of pure Amox produced clear inhibition zones (60.6 ± 1.0 mm for MSSA and 20.6 ± 1.0 mm for MRSA). It could be noted that previous studies successfully demonstrated the antibacterial effects of Amox-loaded electrospun matrices prepared using non-acidic polymer solutions. For example, Zheng et al. developed 0.5–2 wt% Amox-loaded electrospun nano-hydroxyapatite/poly(lactic-*co*-glycolic acid) nanofibers using a THF/DMF solvent system [33]. Similarly, 1–1.5 wt% Amox-loaded polycaprolactone electrospun membranes were fabricated in a chloroform/methanol mixture, showing effective antibacterial activity [34]. Although Amox is generally stable at acidic pH [35], neutral or organic solvents may better preserve its stability during electrospinning process.

**Figure 3:**
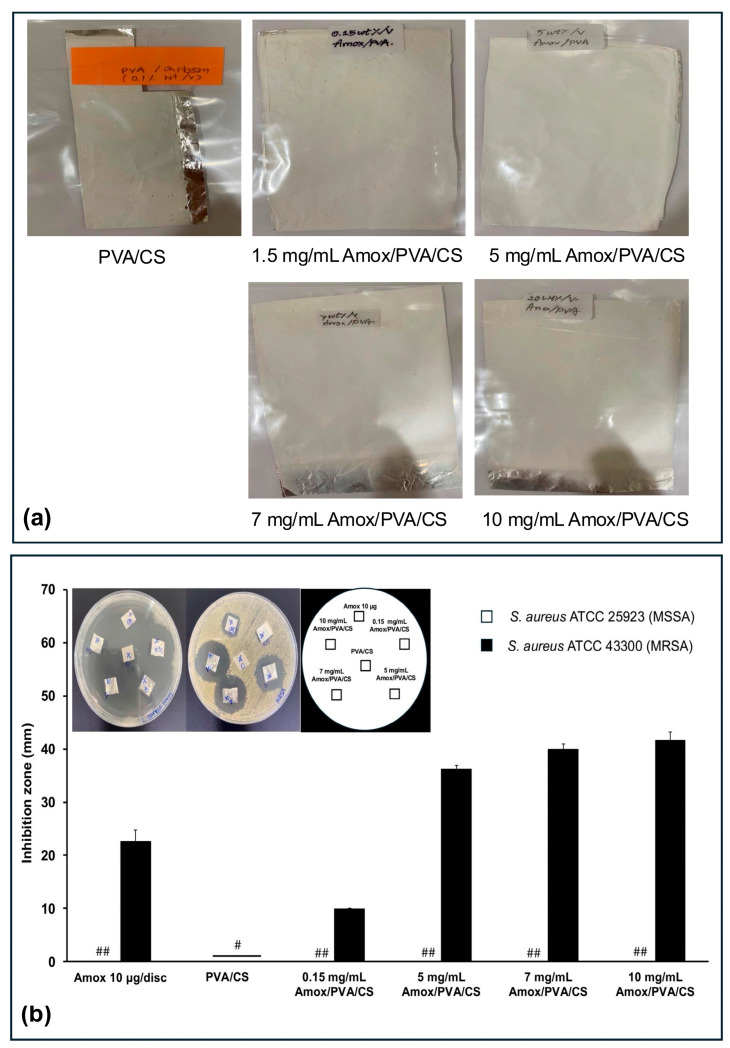
(**a**) Images of the electrospun nanofiber mat fabricated under optimization conditions without acetic acid conditions (formulation set B) and (**b**) their antibacterial activities compared with neat PVA/CS against *S. aureus* ATCC 25923 (MSSA) and 43300 (MRSA). Each symbol indicates the mean ± S.D. (*n* = 3). # The inhibition zone could not be determined. ## A large and overlapping inhibition zone was observed.

In formulation set B, the PVA/CS solution was prepared in DI water without 20% *v/v* acetic acid before incorporating Amox at concentrations ranging from 0.15 mg/mL to 10 mg/mL. All fabricated Amox/PVA/CS mats exhibited a white, opaque appearance (Figure 3a). Figure 3b shows the antibacterial activity of various concentrations of Amox/PVA/CS mats against MSSA and MRSA. For MSSA, the agar plates showed clear zones over most of the area, with bacterial colonies observed near the edges of the Petri dishes. This result indicates that all Amox/PVA/CS mats produced large and overlapping inhibition zones, making individual clear zones difficult to measure. However, the inhibition zones for Amox/PVA/CS mats were measurable against MRSA. The average clear zone diameter increased from 10.0 ± 0.0 mm to 41.7 ± 1.5 mm as the Amox concentration increased from 0.15 mg/mL to 10 mg/mL, suggesting a concentration-dependent efficacy. In the control set, blank PVA/CS mats showed no antibacterial activity, while 10 μg/disc of Amox produced a 22.7 ± 2.1 mm inhibition zone. Notably, Amox/PVA/CS mats containing 0.15 mg/mL (150 μg/mL) Amox prepared without acetic acid retained antibacterial activity, whereas mats containing 250 μg/mL Amox prepared with acetic acid showed no activity. These findings suggest that using acetic acid may degrade Amox and reduce its antibacterial efficacy during electrospinning. The chemical structure of Amox contains a thiazolidine ring fused with a ring of β-lactam and an amino group side chain, which enhances acid stability compared to penicillin [36]. There are three main functional groups—COOH (pKa = 2.7), NH_2_ (pKa = 7.4), and OH (pKa = 9.6)—that are affected by pH changes [36]. The degradation rate of Amox exhibits a U-shaped pH dependence, showing greater stability at pH values close to neutral compared to high acidic or basic environments [37]. Freitas et al. demonstrated that Amox was most stable at pH 5 when incubated at 37 °C for 24 h and the stability decreases significantly at pH 1 and pH 10, with further degradation observed across the pH range as the temperature increases to 55 °C [38]. The degradation of Amox involves the opening of the β-lactam ring, converting it into amoxicilloic acid, which lacks antibacterial activity [36,38]. This may explain the lack of antibacterial activity of Amox/PVA/CS when acetic acid was used as co-solvent (formulation set A).

**Figure 4:**
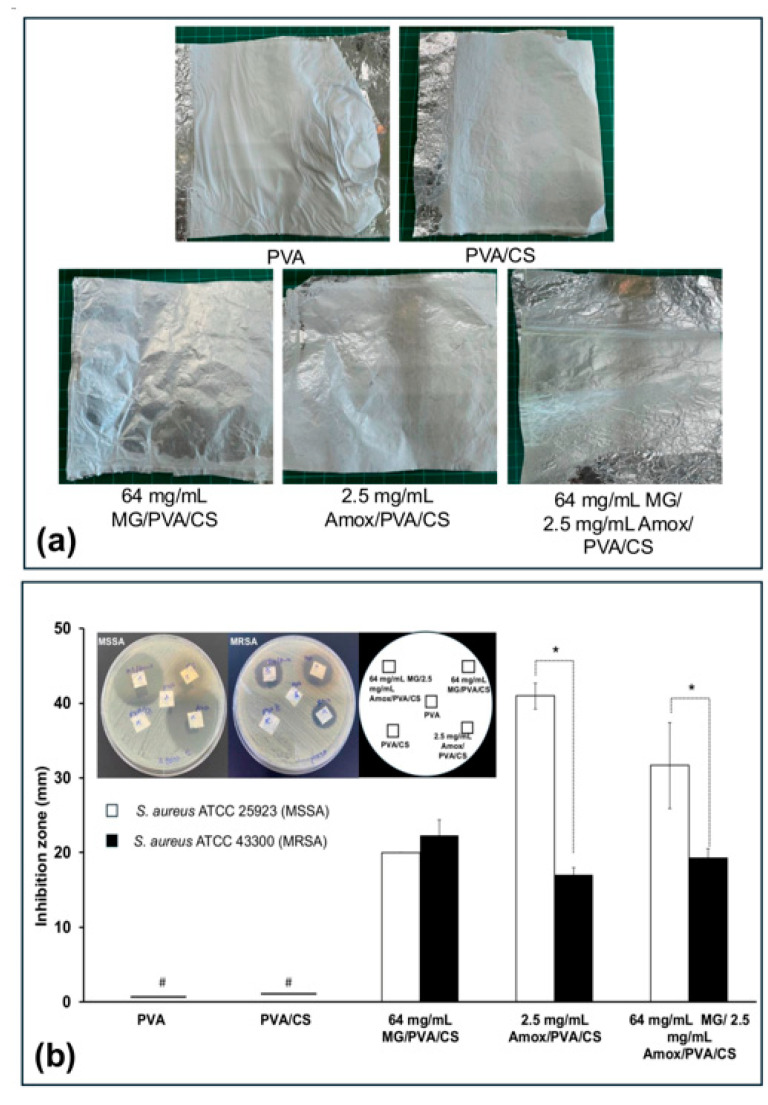
(**a**) Images of the electrospun nanofiber mat and (**b**) the antibacterial activities of the formulation set C; 64 mg MG/PVA/CS, 25 mg Amox/PVA/CS, and 64 mg MG/25 mg Amox/PVA/CS nanofiber mats, compared with neat PVA and PVA/CS against *S. aureus* ATCC 25923 (MSSA) and 43300 (MRSA). Each symbol indicates the mean ± S.D. (*n* = 3). # The inhibition zone could not be determined. * Significant difference between the mean inhibition zones of the two bacterial strains, *p* < 0.05.

In formulation set C, the PVA/CS solution was prepared in DI water to load 64 mg/mL MG and 2.5 mg/mL Amox, aiming to enhance antibacterial activity. As shown in Figure 4a, the fabricated mats had a white, opaque appearance. For antibacterial activity (Figure 4b), neat PVA and PVA/CS showed no inhibition zones against either MSSA or MRSA, suggesting a lack of antibacterial activity in the neat nanofiber mats. The anti-MSSA efficacy followed the order: Amox/PVA/CS > MG/Amox/PVA/CS > MG/PVA/CS. In contrast, the anti-MRSA efficacy followed the order: MG/PVA/CS > MG/Amox/PVA/CS > Amox/PVA/CS. These findings suggest that antibacterial activity is directly derived from the mechanisms of MG or Amox, and the combining MG and Amox in PVA/CS offers greater potential for controlling MRSA infections than using Amox alone. However, the observed anti-MRSA efficacy differed from our previous report, indicating that the mat characteristics may influence antibacterial performance. To better understand these differences, nanofibers from formulation set C were further analyzed for physical, chemical, and thermal properties, as well as swelling and drug release behavior, to explain their antibacterial activity.

### 3.2. Morphology of the Optimized Formulation Set C

**Figure 5:**
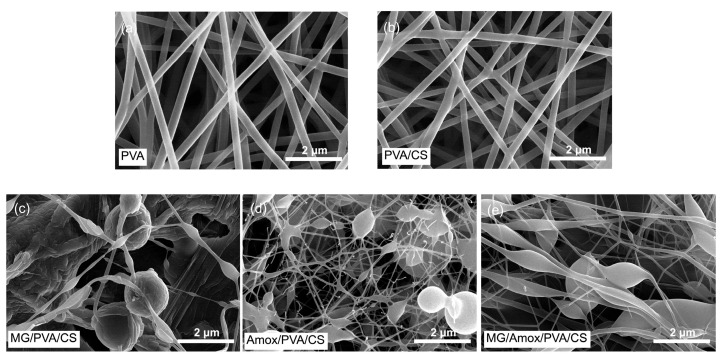
SEM images of fabricated mats from formulation set C: (**a**) PVA, (**b**) PVA/CS, (**c**) MG/PVA/CS, (**d**) Amox/PVA/CS, and (**e**) MG/Amox/PVA/CS.

Table 2 and Figure 5 present the average diameters and morphologies of nanofibers, respectively, for PVA/CS loaded with active compounds. The results show that the neat nanofibers, both PVA and PVA/CS, were bead-free, with the average diameter of PVA fibers (282.0 ± 0.5 nm) being slightly larger than that of neat PVA/CS fibers (256 ± 4 nm). Among the formulations, Amox/PVA/CS exhibited the smallest fiber diameter at 47.3 ± 6.3 nm, followed by MG/PVA/CS (107 ± 15 nm) and MG/Amox/PVA/CS (126 ± 45 nm). However, after loading with active compounds, prominent micron-sized oval and spherical beads were observed. These bead morphologies are in accordance with a previous report after loading 0.1 g rutin and 0.1 g ciprofloxacin in PVA/CS [39]. The formation of beads during electrospinning is a common phenomenon influenced by both solution properties and process parameters. Türkoğlu et al. observed a mix of smooth and beaded fibers when the tip-to-collector distance for a 10% *wt*/*v* PVA solution was reduced from 20 to 5 cm [19]. Generally, a shorter distance between the syringe tip and collector leads to incomplete solvent evaporation, causing partially solvent-swollen fibers to collapse, resulting in non-uniform fibers with larger diameters or beads [40]. When process parameters are fixed, bead formation is primarily affected by solution properties. For example, varying the CS/PVA concentration from 4% to 8% increased the solution viscosity from 2.45 to 20.0 Pa-s [41]. The 4% CS/PVA solution, with its lower viscosity, produced ultrafine fibers with spindle-like beads due to insufficient polymer chain entanglement [41]. Moreover, the shape of the beads shifted from spherical to spindle-like as the polymer concentration and viscosity increased from low to high levels [42]. In our study, with fixed electrospinning process parameters and a constant PVA/CS polymer concentration, bead formation was primarily influenced by drug loading.

Incorporating 64 mg/mL of MG into the fixed-concentration PVA/CS polymer produced larger spherical beads (1.7 ± 0.1 μm) and smaller oval beads (0.33 ± 0.07 μm) compared to the incorporation of 2.5 mg/mL of amoxicillin (Figure 5c,d). These results suggest that higher drug concentrations lead to larger spherical beads. However, when the PVA/CS polymer was loaded with both MG and Amox (Figure 5e), only oval beads were observed, with a diameter of 0.95 μm, indicating partial elongation of the jet during the electrospinning process.

Spherical beads are formed when surface tension dominates, pulling the solution into its lowest-energy configuration—a sphere. In contrast, spindle-like or oval beads result from partial jet elongation, where the viscoelastic forces resist but surface tension still influences the final shape [32,43]. Therefore, higher drug concentrations may increase viscosity and modify surface tension, explaining the bead formation observed in this study.

### 3.3. FTIR Analysis

**Figure 6:**
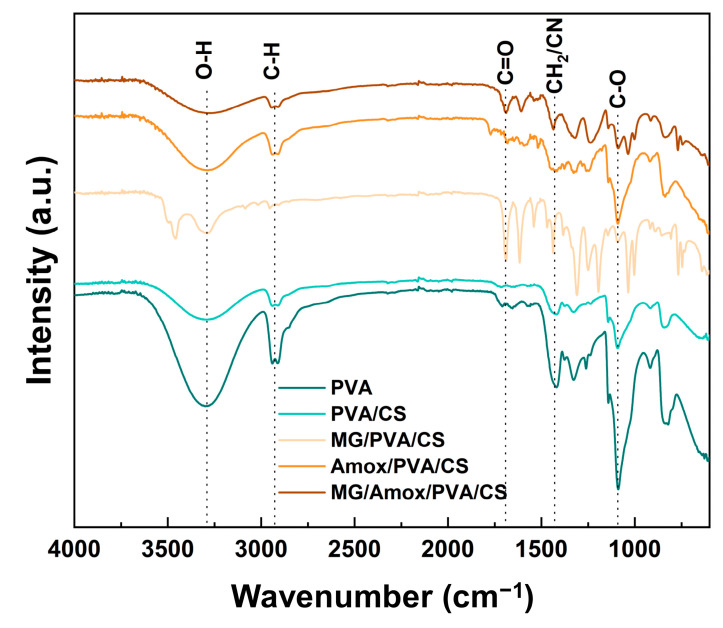
FTIR spectra of PVA, PVA/CS, MG/PVA/CS, Amox/PVA/CS, and MG/Amox/PVA/CS nanofibers from formulation set C.

The FTIR spectra of all electrospun nanofibers are illustrated in Figure 6. For PVA fibers, the characteristic peaks corresponding to the functional groups of pure PVA are clearly observed. A broad absorption peak at 3300–3500 cm^−1^ corresponds to the O–H stretching vibration [44]. Peaks from aliphatic C–H stretching vibrations are observed at 2944 cm^−1^, typical of the PVA backbone [45]. A strong peak at 1090 cm^−1^ is attributed to the C–O stretching vibration in PVA [46].

The blending of CS polymer with PVA slightly modifies the PVA spectrum. Absorption peaks in the 3300–3500, 1421, and 1090 cm^−1^ regions exhibit reduced intensity compared to those of PVA fibers. Moreover, the peaks at 3300–3500 cm^−1^ and 1420 cm^−1^ also indicate N–H stretching and C–N stretching, respectively, which are characteristic of the additional amine and amide groups from CS [44,47].

The incorporation of MG into the PVA/CS fibers displays a more pronounced peak at 1690 cm^−1^, attributed to the stretching vibration of the carbonyl group (–C=O) in the aromatic MG molecule, similar to that observed in MG/Amox/PVA/CS [48]. Moreover, the incorporation of MG enhances the intensity of the peak at 1420 cm^−1^, attributed to CH₂ (aromatic ring) bending vibrations in MG [48]. Although MG/PVA/CS exhibits the hydroxyl absorption peak at 3300–3500 cm^−1^, its intensity is lower than that of PVA, suggesting intermolecular interactions between the hydroxyl groups in the PVA/CS matrix and the functional groups of MG.

For Amox/PVA/CS, a noticeable broad peak was observed at 3300–3500 cm^−1^, attributed to N–H stretching related to the β-lactam and amine groups in Amox. Moreover, the peak at 1090 cm^−1^ exhibits enhanced intensity, likely caused by interactions between the carboxyl group in Amox and the PVA/CS matrix. Similarly, the spectrum of MG/Amox/PVA/CS reflects the combined effects of MG and Amox, with significant enhancements in the 1690 cm^−1^ region (–C=O, carbonyl group) and the 1420 cm^−1^ region (C–N from amine groups) [48]. FTIR analysis confirmed molecular interactions between the loaded drugs and the PVA/CS fibers, highlighting the stability of the drugs within the polymer matrix.

### 3.4. DSC Analysis

**Figure 7:**
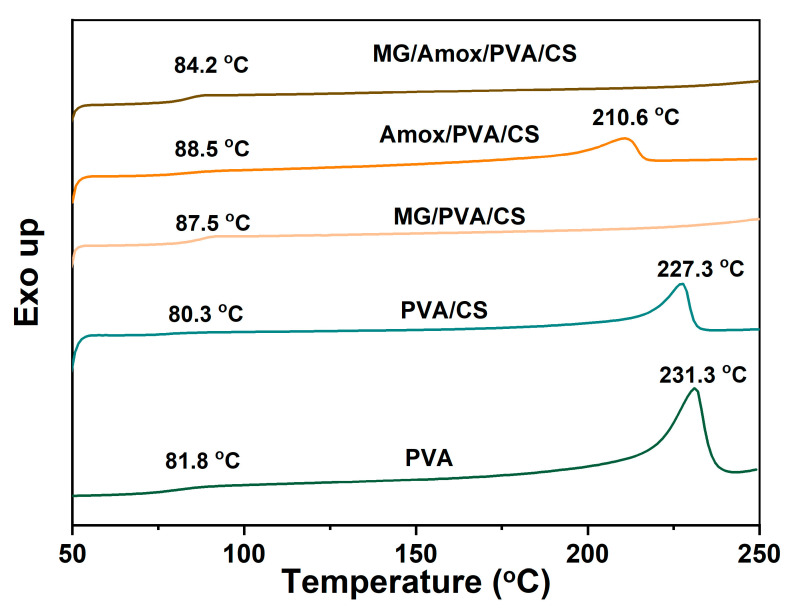
DSC thermograms of PVA, PVA/CS, MG/PVA/CS Amox/PVA/CS, and MG/Amox/PVA/CS nanofibers from formulation set C (heating rate 5 °C/min).

The DSC thermograms illustrate the thermal behavior of the PVA/CS-based nanofiber composites. As shown in Figure 7, the glass transition temperature (T_g_) value was inferred from the first endothermic peaks. The T_g_ of all samples ranged between 80 °C and 88 °C. For PVA, its T_g_ was observed at 81.8 °C. This value is consistent with the typical T_g_ of neat PVA, which generally occurs around 80–85 °C [49]. The second endothermic peak, corresponding to the melting temperature (T_m_) observed in neat PVA, was 231.3 °C, which is associated with the crystalline phase of the PVA polymer. Blending CS with PVA slightly decreased the T_g_ to 80.3 °C, possibly due to reduced crystallinity. This reduction occurs because the amine and amide functional groups of CS can disrupt the dense hydrogen-bonding network of PVA, leading to a slight T_g_ reduction [18,50]. Additionally, the T_m_ of PVA/CS also decreased slightly to 227.3 °C, indicating partial disruption of the PVA crystalline structure by CS, which reduced the thermal stability [50].

In contrast, the incorporation of one or both active compounds (MG and/or Amox) increased the T_g_ by at least 3.9 °C. This increase suggests enhanced intermolecular interactions between the active compounds and the PVA/CS matrix. For example, MG (via its hydroxyl groups) and Amox (via its amine groups) can form hydrogen bonds with the polymer network, stabilizing the matrix and increasing the T_g_ [51]. The increasing T_g_ after incorporating MG could be observed in our previous cellulose acetate blended PVA [25]. However, the T_g_ of MG/Amox/PVA/CS composite decreased slightly to 84.2 °C, compared to samples containing a single active compound (87.5 °C and 88.5 °C for T_g_ of MG/PVA/CS and Amox/PVA/CS, respectively). This slight reduction suggests that competitive interactions among MG, Amox, and the PVA/CS matrix weaken the hydrogen-bonding network, resulting in a more flexible polymer structure. Additionally, MG/PVA/CS and MG/Amox/PVA/CS fibers exhibited no sharp melting peaks. This result suggests a significant reduction in crystallinity, likely due to strong intermolecular interactions between MG and the polymer matrix, which disrupt the ordered crystalline structure.

### 3.5. Swelling, Water Retention, and Weight Loss Behaviors

**Figure 8:**
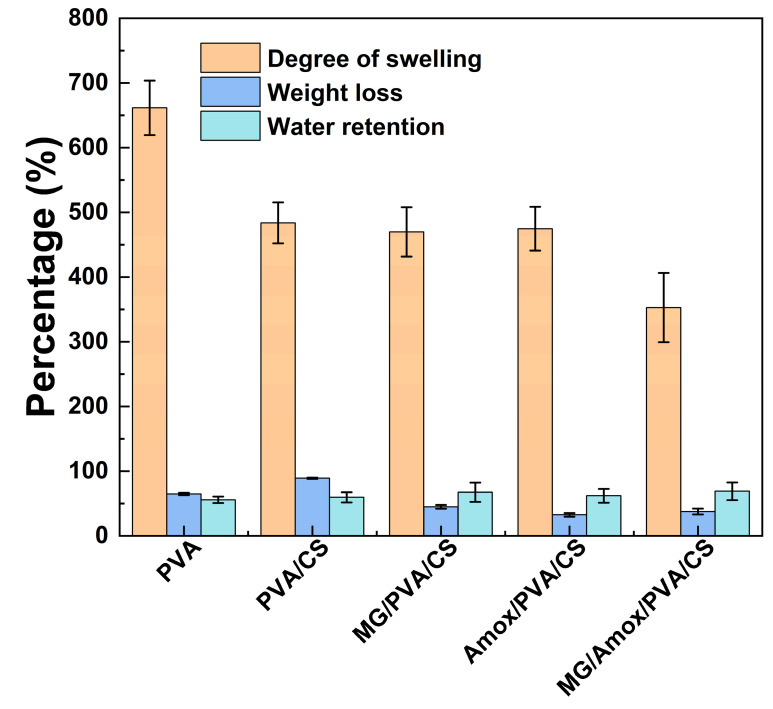
Percentage of swelling degree, weight loss, and water retention of PVA, PVA/CS, MG/PVA/CS, Amox/PVA/CS, and MG/Amox/PVA/CS nanofiber from formulation set C. Data shown as mean ± S.D. (*n* = 3).

As displayed in Figure 8, all formulations exhibited high swelling in DI water for 24 h. The neat PVA fibers showed the highest swelling of 661.6 ± 42.1%, indicating that pure PVA has the greatest water absorption capacity due to its hydrophilic hydroxyl groups and minimal structural restrictions. When CS was blended with PVA (PVA/CS), the degree of swelling slightly decreased to 483.6%. This reduction may have been caused by hydrogen bonding interactions between PVA and CS, which reduce the availability of free hydroxyl groups to absorb water [52]. However, the incorporation of single compounds further slightly decreased swelling with less than 13.8% reduction compared to PVA/CS. Moreover, incorporating both MG and Amox decreased swelling further with the swelling percentage of 352.8 ± 53.4%, indicating their interaction with the polymer matrix, possibly forming cross-linked structures or restricting water uptake. A large surface-area-to-mass ratio of the fiber mat is one of the important factors regarding the degree of swelling due to higher water retention [53]. Edikresnha et al. demonstrated that electrospun polyvinylpyrrolidone/cellulose acetate composite nanofibers loaded with glycerin and garlic extract exhibited a significant reduction in swelling—over 78%—compared to the neat electrospun polymer. This decrease was attributed to the larger fiber diameters and reduced porosity of the fibrous mat [54]. In this study, SEM images revealed that MG/Amox/PVA/CS mats exhibit larger fiber and oval bead diameters compared to MG/PVA/CS or Amox/PVA/CS mats. This increase in size reduces the contact area between the fibers and water, resulting in lower liquid absorption and degree of swelling.

The water retention capacity across all formulations ranged from 55.7% to 68.9%, with the following trend: MG/Amox/PVA/CS > MG/PVA/CS > Amox/PVA/CS > PVA/CS > PVA. The total water retained within the fiber mats is attributed to a combination of water absorbed into the porous structure and the fiber matrix itself, which can be influenced by fiber diameter [55]. Smaller fiber diameters typically result in a higher surface area-to-volume ratio, leading to increased porosity [55]. Consequently, this could explain the higher water retention observed in neat fiber mats PVA and PVA/CS compared to composite fiber loading with active compounds. However, Amox/PVA/CS, despite exhibiting the smallest fiber diameter of approximately 47 nm, demonstrated lower water retention compared to MG/Amox/PVA/CS and MG/PVA/CS. It is noteworthy that this result contradicts the trend of increasing T_g_ in composite materials. This finding suggests that enhanced molecular interactions resulting from chemical bonding with CS, MG, and Amox contribute to reduced water uptake. Therefore, this can explain the lowest water retention of Amox/PVA/CS, which has the highest Tg among the composite fibers.

Typically, weight loss is more pronounced in materials with higher porosity and smaller fibers due to their increased susceptibility to degradation. As shown in Figure 8, the weight loss (%) followed the order PVA/CS > PVA > MG/PVA/CS > MG/Amox/PVA/CS > Amox/PVA/CS. The neat fiber mat exhibited a higher weight loss (>64.8%), whereas fiber mats loaded with active compounds demonstrated lower weight loss (<44.8%). These findings suggest that the addition of MG and Amox improved the structural stability of PVA/CS by increasing intramolecular bonding. This enhancement in stability is supported by the FTIR results, which indicate stronger interaction, and the DSC data, which show increased thermal stability.

### 3.6. Release Behaviors

**Figure 9:**
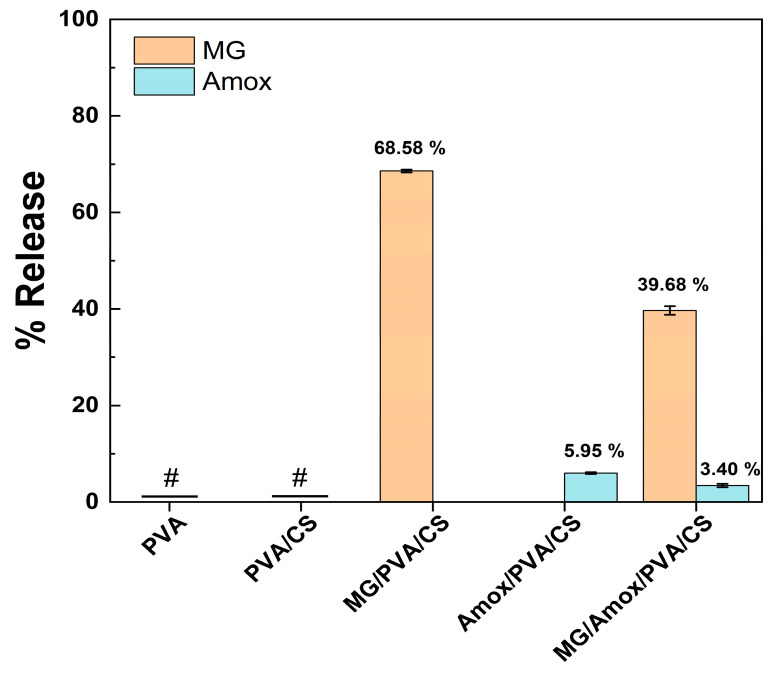
The 24 h percentage release of MG and Amox from PVA/CS electrospun (formulation set C). Data shown as mean ± S.D. (*n* = 3). # The concentration of MG and Amox could not be determined.

The 24 h release of MG and Amox was analyzed to explain the anti-*S. aureus* activity observed after applying the fiber mat to a bacterial agar plate for 24 h. As shown in Figure 9, the release of MG and Amox from the PVA/CS nanofiber mat into DI water was measured over the same period. For the neat PVA and PVA/CS samples without active compounds, no detectable MG or Amox was observed in the release solution, confirming the absence of these compounds in the respective formulations. For the PVA/CS nanofiber mat loaded with a single active compound, MG was released at approximately 68.58%, while the release of Amox was significantly lower at only 5.95%. Because Amox is a poor water soluble drug of 2.7 mg/mL [56], the hydrophobicity of Amox may explain the low drug release from the PVA/CS mat. This result agreed with previous study of Sofokleous et al., who studied the release of Amox from poly(D,L-lactide-*co*-glycolide) (PLGA) electrospun fiber [57]. The drug release from the PLGA electrospun fiber was found to be lower than 10% within 24 h in distilled water and reach 68% within 21-day release period [57]. In the case of the MG/Amox/PVA/CS composite, the release percentages of both MG and Amox were significantly reduced by approximately 42% compared to the single-compound-loaded samples, possibly due to their low degree of swelling. Additionally, MG/Amox/PVA/CS mats with larger fiber and bead diameters led to a smaller surface area for compound release compared to single-compound-loaded PVA/CS mats. Additionally, the larger fiber and bead diameters of the MG/Amox/PVA/CS mats result in a smaller surface area for compound release compared to single-compound-loaded PVA/CS mats. These characteristics explain the differences in anti-MRSA efficacy of MG and Amox observed between the in vitro study and the fiber-formulated mats.

## 4. Conclusions

MG (64 mg/mL) and Amox (2.5 mg/mL) were successfully incorporated into 0.1% *wt*/*v* CS and 10% *wt*/*v* PVA nanofibers via electrospinning. The acidity of the polymer solution was found to be a critical factor influencing the antibacterial activity of Amox during the electrospinning process. To optimize the formulation, MG/Amox/PVA/CS nanofibers were prepared in DI water, resulting in white fiber mats. However, the anti-*S. aureus* activity of MG/Amox/PVA/CS nanofiber mats showed no significant improvement over MG or Amox alone against MRSA, likely due to the physicochemical characteristics of the fabricated fibers. Among the samples, MG/Amox/PVA/CS revealed nanosized fibers with the largest fiber and micron-sized bead structures, and FTIR and DSC analysis confirmed molecular interactions between the active compounds and the PVA/CS matrix. Moreover, it exhibited the least swelling properties and lowest release percentages at 24 h. This study provides valuable insights into the relationship between the characteristics of MG/Amox-loaded PVA/CS nanofibers and their anti-*S. aureus* applications. However, this research is limited to in vitro testing against two *S. aureus* isolates, which may not reflect clinical applicability. Further studies on clinical bacterial isolates, in vitro cytotoxicity, in vivo wound healing efficacy, and nanofiber stability are needed to validate their medical potential.

## Figures and Tables

**Table 1:** The concentrations of polymers and active compounds in formulation sets A, B, and C.

**Formulation set A:**					
**Concentrations (mg/mL with acetic acid)**					
	PVA	PVA/CS	MG/PVA/CS	Amox/PVA/CS	MG/Amox/PVA/CS
**PVA**	100	100	100	100	100
**CS**	0	1	1	1	1
**Amox**	0	0	0	0.25 (0.25 wt%)	2.5
				or 1 (1 wt%)	
				or 4 (4 wt%)	
**MG**	0	0	64	0	64
**Formulation set B:**					
**Concentrations (mg/mL without acetic acid)**					
	PVA	PVA/CS	MG/PVA/CS	Amox/PVA/CS	MG/Amox/PVA/CS
**PVA**	-	100	-	100	-
**CS**	-	1	-	1	-
**Amox**	-	0	-	0.15 (0.15 wt%)	-
				or 5 (5 wt%)	
				or 7 (7 wt%)	
				or 10 (10 wt%)	
**MG**	-	0	-	-	-
**Formulation set C:**					
**Concentrations (mg/mL without acetic acid)**					
	PVA	PVA/CS	MG/PVA/CS	Amox/PVA/CS	MG/Amox/PVA/CS
**PVA**	100	100	100	100	100
**CS**	0	1	1	1	1
**Amox**	0	0	0	2.5	2.5
**MG**	0	0	64	0	64

**Table 2:** Diameters of PVA/CS nanofiber loading of MG and Amox.

Formulation Set C	Average Diameter		
	Fibers (nm)	Oval Bead (μm) *	Spherical Bead (μm) *
PVA	282.0 ± 0.5	-	-
PVA/CS	256 ± 4	-	-
MG/PVA/CS	107 ± 15	0.33 ± 0.07	1.7 ± 0.1
Amox/PVA/CS	47.3 ± 6.3	0.39 ± 0.01	1.3 ± 0.3
MG/Amox/PVA/CS	126 ± 45	0.95 ± 0.4	-

* Density of oval and spherical beads could not be determined under layers of fibers.

## Data Availability

The data are contained within the article.
